# Editorial: Neuroimaging in Veterinary Science

**DOI:** 10.3389/fvets.2019.00217

**Published:** 2019-07-04

**Authors:** Andrea Tipold, Fintan J. McEvoy

**Affiliations:** ^1^Department of Small Animal Medicine and Surgery, University of Veterinary Medicine Hannover, Hanover, Germany; ^2^Department of Veterinary Clinical Sciences, University of Copenhagen, Copenhagen, Denmark

**Keywords:** animal models, MRI - magnetic resonance imaging, FLAIR (fluid attenuated inversion recovery), SPECT (single photon emission tomography), PET - positron emission tomography, dynamic MRI, MRI muscle imaging, dynamic susceptibility contrast (DSC)

This collection of 15 papers focuses on the developments in Neuroimaging in Veterinary Science over the last couple of years. It sheds light on aspects of current understanding and the role of imaging in important neurological diseases affecting veterinary patients and their caregivers and on areas of related research. Advances in neuroimaging have changed clinical neurology not only by contributing to improved understanding of relevant pathophysiology, but also by describing new diseases or new features of well-known diseases. This research topic places current work in veterinary neuroimaging in the context of our current understanding of clinical signs and the pathophysiology of diseases. It points a way toward future trends in the field.

There are many ways to look at the collection, depending on the specific background of the reader. The papers are mainly concerned with MRI but comparison is made with CT with regard to oncology imaging. The anatomical regions examined are as expected, brain and spinal cord including the vertebral column, but also there is consideration of the changes in para-spinal muscle groups in their response to intervertebral disc herniation. Neurological diseases are covered from the clinical diagnosis, pathophysiology and imaging protocol points of view.

With regard to spinal cord diseases, the research review contribution by Rusbridge et al. highlights research concerned with Chiari like malformation and syringomyelia, the role of syringomyelia (SM) in symptomatic disease is discussed and criteria are suggested for SM evaluation. The research paper by Kunze et al. describes the discrepancy between the grading of intervertebral disc degeneration according to the particular MRI sequence chosen. It is a reminder of the flexibility of MRI as an imaging tool, and that different sequences yield different, often non-overlapping information with respect to the anatomy and pathology imaged. In an anatomical study by Düver et al. disk to vertebral body dimension ratios are reported and breed specific differences emphasized. As a result, the understanding of breed specific diseases of the vertebral column can be improved. Again with respect to intervertebral disc disease, the paper by Trampus et al. describes alterations in MR signal from muscles in dogs with thoracolumbar disc disease; focal hyperintense T2W signals are described in muscle at sites of intervertebral disc disease.

Diseases of the brain and cranial cavity feature strongly in this research collection. The review paper by Rusbridge et al. considers MRI features suggestive of raised intracranial pressure in patients with ventriculomegaly. It identifies important anatomical abnormalities such as rostral displacement of the axis and atlas, and increased angulation of the odontoid process that should be considered in the search for causes of cerebrospinal fluid flow disruption. It also suggests novel MRI sequences to assist in the detection of adhesions and arachnoid webs in the subarachnoid space. Anatomical malformations are also considered in the research reported by Lauda et al. In their paper the caudal fossa ratio in normal dogs is compared with the same ratio in Eurasier dogs with VLDLR (very low density lipoprotein receptor) -associated genetic cerebellar hypoplasia. They report that a sub-population of Eurasier dogs with this genetic defect show altered ratios, but not all. This suggests two phenotypes for the single genetic defect.

Physiology and pathophysiology of the brain and of intracranial diseases, detected by MRI are considered by two research papers. In the paper by Schmidt et al. perfusion magnetic resonance imaging is used to demonstrate reduced periventricular blood flow in dogs with ventriculomegaly. In the research paper by Moioli et al. changes in the intensity of the MR signal on T2 weighted fluid-attenuated inversion recovery sequences are reported to be a function of the fraction of inspired oxygen. As signal strength from CSF on these sequences is used in the evaluation of pathology, the possibility of this physiological cause of signal alteration is important.

Neoplasia, epilepsy and inflammatory brain disease also feature in the collection. In the paper by Stadler et al. a comparison is made between the accuracy of CT and MRI in determining glioma type (astrocytoma or oligodendroma), and glioma grade, high (WHO grade III or IV) or low (WHO grade II). Both modalities showed similar diagnostic performance in this clinical scenario, but the authors identified that both CT and MRI have many facets, and that the addition of techniques such as dynamic contrast—enhanced imaging in CT, or spectroscopy, or diffusion weighted imaging in MR may allow improvement in diagnostic performance.

Epilepsy is a common indication and a research focus for neuroimaging. In the review paper by Bankstahl and Bankstahl the current role of nuclear medicine in the veterinary epilepsy patient is discussed and its future role in diagnosis and research suggested. While PET and SPECT scanners are not commonly available in the wider clinical veterinary community they are not infrequently accessible in larger research settings. This paper suggests that there is value in continued use of nuclear imaging modalities in this patient group. The publication by Estey et al. identified volumetric changes in dogs with idiopathic epilepsy. With such studies it becomes evident that seizures have an impact on the brain structure or that some cases with presumed idiopathic epilepsy may have a structural cause for occurring seizures that are only detectable by advanced MR imaging and special analysis.

Inflammatory brain disease in the form of necrotizing encephalitis in dogs is reviewed in the paper by Flegel. The paper provides a helpful guide to MR imaging characteristic of necrotizing encephalitis in dogs and suggests that breed-specific imaging characteristics allow a clinical and imaging diagnosis with a relatively high degree of certainty. Flegel summarizes typical breed specific imaging features of the disease, such as lesion distribution, signal intensity, contrast enhancement, and gross changes of brain structure.

The collection also comprises papers illustrating the value of individual case reports. The paper by De Decker et al. reports imaging findings in a patient with tethered cord syndrome and highlights the potential usefulness of comparing images of the lumbosacral region obtained in neutral, flexed and extended positions to demonstrate an expected displacement of the *conus medullaris*. This so called dynamic lumbosacral MRI was of use in the patient reported and its routine application is suggested for further evaluation. The case report by Wang-Leandro et al. shows a new clinical feature of an inflammatory disease of the meninges leading to hyperacute hemorrhage. MRI features of such an early stage of hemorrhage are rarely described because of the time delay between occurrence of clinical signs and the diagnostic workup in MRI.

New ways to extract and measure data from the complicated medical imaging devices available to us is always of interest. In the research paper by Stadler et al. protocols for dynamic susceptibility contrast MRI of the canine brain are presented. These protocols (similar but with differing contrast dose per kg according to body weight) allow the creation of perfusion maps of the canine brain. These will undoubtedly have relevance for clinical veterinary neurology. Differences in size and shape amongst veterinary patients also feature in the paper by Pilegaard et al. which examines the influence of cranial shape on the ratio of the volume of the lateral ventricles to that of the cerebral cortex. It cautions that skull shape influences this index of relative volumes and so the index can be seen as breed dependent.

In setting up this Frontiers Research Topic we hoped to receive input that covered a wide range of topics in veterinary neuroimaging in the expectation that the resulting collection of work would be source of reference, information and inspiration. The collected work achieves this. It is the work of many individuals and it highlights both how much imaging has contributed and can be expected to contribute to veterinary neuroscience.

## Author Contributions

All authors listed have made a substantial, direct and intellectual contribution to the work, and approved it for publication.

### Conflict of Interest Statement

The authors declare that the research was conducted in the absence of any commercial or financial relationships that could be construed as a potential conflict of interest.

